# Supplementary material for: Efficacy of Artificial-Intelligence-Driven Differential-Diagnosis List on the Diagnostic Accuracy of Physicians: An Open-Label Randomized Controlled Study
Source: Int J Environ Res Public Health. 2021 Feb 21;18(4):2086. doi: 10.3390/ijerph18042086 (PMC7924871; doi:10.3390/ijerph18042086)
Supplement: Supplementary file 1 [file ijerph-18-02086-s001.zip › suppl/20210126_IJERPH_UbieDDx_RCT_suppl.docx]

**Table S1. Diagnostic accuracy in individual cases**

| **Case number** | **With AI-driven differential diagnosis list** | **Without AI-driven differential diagnosis list** | ***P*-value** |
| --- | --- | --- | --- |
| **1** | 6/11 (54.5%) | 8/11 (72.7%) | >0.66* |
| **2** | 11/11 (100%) | 10/11 (90.9%) | >0.99* |
| **3** | 5/11 (45.5%) | 8/11 (72.7%) | 0.33* |
| **4** | 11/11 (100%) | 9/11 (81.8%) | 0.48* |
| **5** | 7/11 (63.6%) | 8/11 (72.7%) | >0.99* |
| **6** | 0/11 (0.0%) | 0/11 (0.0%) | >0.99* |
| **7** | 10/11 (90.9%) | 9/11 (81.8%) | >0.99* |
| **8** | 2/11 (18.2%) | 4/11 (36.4%) | 0.64* |
| **9** | 0/11 (0.0%) | 1/11 (9.1%) | >0.99* |
| **10** | 0/11 (0.0%) | 0/11 (0.0%) | >0.99* |
| **11** | 5/11 (45.5%) | 5/11 (45.5%) | >0.99* |
| **12** | 11/11 (100%) | 11/11 (100%) | >0.99* |
| **13** | 9/11 (81.8%) | 6/11 (54.5%) | >0.36* |
| **14** | 11/11 (100%) | 10/11 (90.9%) | >0.99* |
| **15** | 2/11 (18.2%) | 0/11 (0.0%) | 0.48* |
| **16** | 11/11 (100%) | 10/11 (90.9%) | >0.99* |

*Fisher exact test

**Table S2. Diagnostic accuracy of vignettes in which the AI-driven differential diagnosis list included a correct diagnosis.**

|  | **With AI-driven differential diagnosis list** | **Without AI-driven differential diagnosis list** | ***P*-value** |
| --- | --- | --- | --- |
| **Total** | 74/88 (84.1%) | 64/88 (72.7%) | 0.10 |
| **Sex** |  |  |  |
| **Male** | 46/56 (82.1%) | 55/72 (76.4%) | 0.57 |
| **Female** | 28/32 (87.5%) | 10/16 (62.5%) | 0.06* |
| **Experience** |  |  |  |
| **Intern** | 20/24 (83.3%) | 8/16 (50.0%) | 0.04* |
| **Resident** | 28/32 (87.5%) | 25/32 (78.1%) | 0.51 |
| **attending physician** | 26/32 (81.3%) | 32/40 (80.0%) | >0.99 |
| **Trust AI** |  |  |  |
| **Yes** | 41/48 (85.4%) | 40/56 (71.4%) | 0.14 |
| **No** | 33/40 (82.5%) | 25/32 (78.1%) | 0.63 |

*Fisher exact test

**Table S3. Diagnostic accuracy of the vignettes in which the AI-driven differential diagnosis list did not include a correct diagnosis.**

|  | **With AI-driven differential diagnosis list** | **Without AI-driven differential diagnosis list** | ***P*-value** |
| --- | --- | --- | --- |
| **Total** | 27/88 (30.7%) | 34/88 (38.6%) | 0.34 |
| **Sex** |  |  |  |
| **Male** | 17/56 (30.4%) | 27/72 (37.5%) | 0.51 |
| **Female** | 10/32 (31.3%) | 7/16 (43.8%) | 0.59 |
| **Experience** |  |  |  |
| **Intern** | 3/24 (12.5%) | 4/16 (25.0%) | 0.41* |
| **Resident** | 12/32 (37.5%) | 16/32 (50.0%) | 0.45 |
| **attending physician** | 12/32 (37.5%) | 14/40 (35.0%) | >0.99 |
| **Trust AI** |  |  |  |
| **Yes** | 10/48 (20.8%) | 23/56 (41.1%) | 0.046 |
| **No** | 17/40 (42.5%) | 11/32 (34.4%) | 0.65 |

*Fisher exact test

**Table S4. Omission and commission errors.**

|  | **Omission errors** | **Commission errors** |
| --- | --- | --- |
| **Total** | 14/88 (15.9%) | 26/176 (14.8%) |
| **Male** | 10/56 (17.9%) | 21/112 (18.8%) |
| **Female** | 4/32 (12.5%) | 5/64 (7.8%) |
| **Intern** | 4/24 (16.7%) | 12/48 (25.0%) |
| **Resident** | 4/32 (12.5%) | 8/64 (12.5%) |
| **Attending physician** | 6/32 (18.8%) | 6/64 (9.4%) |
| **Trust AI** | 7/48 (14.6%) | 19/96 (19.8%) |
| **Do not trust AI** | 7/40 (17.5%) | 7/80 (8.8%) |

AI, artificial intelligence
